# *S. cerevisiae* Strain Lacking Mitochondrial IF3 Shows Increased Levels of Tma19p during Adaptation to Respiratory Growth

**DOI:** 10.3390/cells8070645

**Published:** 2019-06-26

**Authors:** Sergey Levitskii, Maria V. Baleva, Ivan Chicherin, Igor A. Krasheninnikov, Piotr Kamenski

**Affiliations:** 1 M.V. Lomonosov Moscow State University, Faculty of Biology, 119234 Moscow, Russia; krolick@yandex.ru (S.L.); mary-bw@mail.ru (M.V.B.); i.v.chicherin@gmail.com (I.C.); iakrasheninnikov@protein.bio.msu.ru (I.A.K.); 2 M.V. Lomonosov Moscow State University, Institute of Functional Genomics, 119234 Moscow, Russia

**Keywords:** mitochondria, mitochondrial translation, Aim23p, Tma19p

## Abstract

After billions of years of evolution, mitochondrion retains its own genome, which gets expressed in mitochondrial matrix. Mitochondrial translation machinery rather differs from modern bacterial and eukaryotic cytosolic systems. Any disturbance in mitochondrial translation drastically impairs mitochondrial function. In budding yeast *Saccharomyces cerevisiae*, deletion of the gene coding for mitochondrial translation initiation factor 3—*AIM23*, leads to an imbalance in mitochondrial protein synthesis and significantly delays growth after shifting from fermentable to non-fermentable carbon sources. Molecular mechanism underlying this adaptation to respiratory growth was unknown. Here, we demonstrate that slow adaptation from glycolysis to respiration in the absence of Aim23p is accompanied by a gradual increase of cytochrome c oxidase activity and by increased levels of Tma19p protein, which protects mitochondria from oxidative stress.

## 1. Introduction

Mitochondria are obligate organelles of the majority of eukaryotic cells; they play a vital role in a large number of metabolic processes, such as energy production, amino acid metabolism, FeS-clusters synthesis, etc. According to the generally accepted endosymbiotic theory, mitochondria have evolved from a prokaryotic ancestor closely related to modern *Rickettsia sp*. [1] During evolution, most of mitochondrial genes were transferred to the nucleus or completely lost. Almost all proteins dispensable for mitochondria functionality are nucleus-encoded, synthesized in cytosol, and imported into mitochondria via specific translocation mechanisms [2]. Nevertheless, modern mitochondria retain a part of their ancestor’s genome, which typically encodes specific mitochondrial ribosomal RNAs, set of tRNAs, and several proteins that are mostly the components of electron transport chain [3]. Besides, mitochondria have their own mechanisms of transcription and protein synthesis [4].

Mitochondrial translation is organized, in general, as in prokaryotes, but has several significant differences, largely due to evolutionary substitution of some ribosomal RNA functions by specific proteins and fine tuning of translation in organelles [5]. One of the stages that significantly differs from those of prokaryotic protein synthesis is mitochondrial translation initiation. Particularly, all studied vertebrates’ mitochondria lack the ortholog of bacterial initiation factor 1. Instead, its functions are implemented by a short domain inserted to mitochondrial IF2 [6]. On the other hand, in invertebrates the functional analog of IF1 has not been identified [7].

Several years ago, using bioinformatic and genetical approaches we identified the previously unknown ortholog of translation initiation factor 3 in *S. cerevisiae* mitochondria, Aim23p [8]. Surprisingly, the presence of mtIF3 ortholog is not necessary for mitochondrial protein synthesis. The deletion of *AIM23* gene leads not to the arrest of mitochondrial translation but rather to the imbalance in mitochondrial protein synthesis [9]. This is reflected in decreased synthesis of Cox1p and Cox2p proteins, which are the components of cytochrome c oxidase, and increased translation of ATP6/ATP8 and ATP9 mRNAs, components of ATP-synthase complex. Moreover, this imbalanced translation does not prevent growth of yeast on media with non-fermentable carbon sources, but just significantly lengthens the lag-phase on growth curve. The wild-type yeasts need about 16–18 h to reach log-phase, while *aim23*Δ strain adapts to respiratory growth for about 50–60 h. Moreover, strain with *AIM23* deletion after long adaptation quickly reaches wild-type optical density values and even exceeds it [9]. This work is aimed at the determination of the processes underlying the adaptation of translationally-imbalanced *aim23Δ* strain.

## 2. Materials and Methods

### 2.1. Strains, Media, and Growth Conditions

Strains used in this work were derivatives of D273-10B DUL2 (MATa mal (lys2, ura3), kindly gifted by Thomax Fox, Cornell University, USA). Genomic disruption of *AIM23* gene was performed using KanMX4 cassette containing geneticin (G418) resistance gene [10]. This cassette was obtained by PCR from pFA6-KanMX4 vector [11] using oligonucleotides that contained 5′- and 3′-proximal parts complementary to the upstream and downstream regions of *AIM23* gene, respectively, for homologous recombination. Yeast haploid strain D273-10B was transformed by the above-described cassette as per [12]. Transformants were selected on the plates with G418. Screening of the G418-resistent clones was performed by PCR. The resulting *aim23*∆ strain was checked for the characteristic phenotype detected earlier [9] (delay of growth on glycerol-containing medium). Disruption of *TMA19* gene was performed in the same manner, by PCR-synthesis of NatNT2 cassette using pFF6-NatNT2 vector [13]. Transformants were selected on the plates with nourseothricin.

For 9-myc-tagging of Tma19p, the DNA fragment containing the myc-tag-coding sequence and NatNT2 cassette flanked by 45 bases *TMA19* homology regions was PCR-amplified. The product was transformed into D2713-10b or *aim23*∆ strains as per [12]. Transformants were selected on the plates with nourseothricin.

Yeasts were grown either in YPD (2% pepton, 1% yeast extract, and 2% dextrose), YPGal (2% pepton, 1% yeast extract, and 2% galactose) or YPEG (2% pepton, 1% yeast extract, 3% glycerol, and 2% ethanol) media.

### 2.2. Mitochondria Isolation

Mitochondria were isolated as described in [14] with minor modifications. Briefly, yeasts were grown in YPEG medium to optical density at 600 nm (OD_600_) ~2, collected by centrifugation at 4000× *g* for 10 min, washed twice with water and resuspended in DTT buffer (0.1 M Tris-H_2_SO_4_, 10 mM DTT) at 2 mL/g wet weight ratio. After 30 min incubation at 30 °C, cells were collected by centrifugation, washed with zymolyase buffer (20 mM K-phosphate buffer pH 7.4 and 1.2 M sorbitol) at 7 mL/g wet weight ratio, and resuspended in the same volume of zymolyase buffer with 1 mg/g Zymolyase 20T. After incubation for 1 h at 30 °C and 80 rpm in orbital shaker, spheroplasts were collected by centrifugation and washed again with zymolyase buffer at the same ratio. Then spheroplasts were resuspended in homogenization buffer (10 mM Tris-HCl pH 7.4, 0.6 M sorbitol, 1 mM EDTA, 1 mM PMSF, and 0.2% BSA) and disrupted in Dounce homogenizer (15 strokes). Lysed spheroplasts were centrifuged at 3000× *g* for 10 min, and crude mitochondrial fraction was sedimentated at 17,000 g for 15 min. The pellet was gently resuspended in minimal volume of SEM buffer (10 mM MOPS-KOH pH 7.2, 250 mM sucrose, and 1 mM EDTA), and then was applied at 15%/23%/32%/60% sucrose step gradient for ultracentrifugation at 134,000× *g* for 1 h. Pure mitochondrial fraction from interphase between 60% and 32% of sucrose was collected, diluted triple in SEM buffer, and sedimentated at 17,000× *g* for 15 min.

### 2.3. Cytochrome c Oxidase Activity Assay

Cytochrome c was reduced as follows. In total, 2.7 mg of cytochrome c was solubilized in 1 mL of water, then 0.1 M DTT solution was added up to 0.5 mM, and the resulting mixture was incubated for 15 min. Cytochrome c reduction was controlled by measuring A_550_/A_565_ ratio, which should be between 10 and 20.

The activity of cytochrome c oxidase was measured as per [15] with minor modifications using Tecan NanoQuant Infinite M200 Pro (Tecan Group Ltd, Männedorf, Switzerland) apparatus in 96-well plates. Reaction was carried out in 200 µL of 10 mM Tris-HCl pH 7.0, 125 mM KCl, and 0.5 mM reduced cytochrome c from equine heart at 37 °C. Approximately 1 µg of mitochondria was added to reaction mixture, and OD_550_ measurement was started immediately and continued for 2 min. Reaction was stopped by the addition of KCN up to 2.5 mM, and OD_550_ was monitored for 1 min after KCN addition. All reactions were performed in triplicates and normalized to mitochondrial protein content.

### 2.4. Blue Native Polyacrylamide Gel Electrophoresis (PAGE)

Blue native PAGE was performed as it described in [16] with minor modifications. About 100 µg of mitochondria were solubilized in 3% digitonin at 1:6 (total mitochondrial protein : digitonin) ratio for 30 min on ice, centrifuged at 30,000× *g* for 30 min, and applied to 4–10% Blue native PAGE. After electrophoresis gels were sliced, the tracks were additionally stained with Coomassie G250 or stained for CIV activity with cytochrome c and diaminobenzidine, as described in [17].

### 2.5. 2D Differential Proteomic Analysis (2D-DIGE)

2D-DIGE with subsequent MALDI-ToF protein identification was performed as per [18] in the Institute of Physico-Chemical Medicine, Moscow, Russia.

### 2.6. Growth Curves

Yeasts from overnight cultures were inoculated in 1 mL of YPEG medium to OD_600_ 0.01 in 24-well plate in 3 replicates. Growth curves were plotted using Tecan NanoQuant Infinite M200 Pro device (Tecan Group Ltd, Männedorf, Switzerland) at 30 °C, 200 rpm for 96 h with OD_600_ measurement every 15 min. Experiments were repeated triple. For antioxidant experiments, N-acetylcysteine and α-tocopherol were added to medium up to 5 mM and 1 mM, respectively.

## 3. Results

### 3.1. Adaptation of aim23Δ Yeast Strain to Respiratory Growth Does Not Completely Restore Cytochrome Oxidase Activity

**Figure 1:**
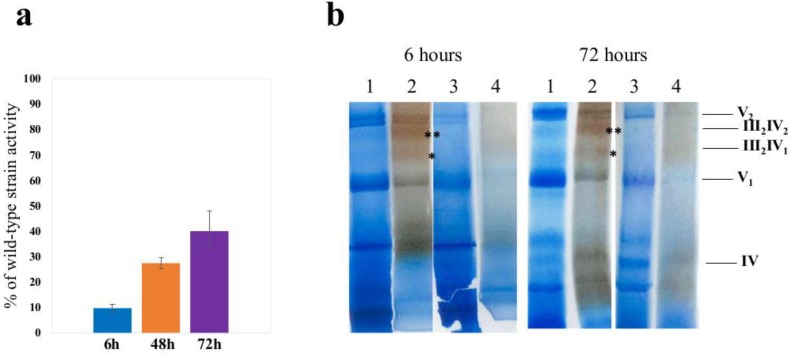
Cytochrome c oxidase activity and supercomplex formation before and after adaptation of yeasts to respiratory growth. (**a**) CIV activity in mitochondria isolated from *aim23Δ* after 6, 48, and 72 h of cultivation on YPEG, expressed in percent of wild-type activity (measured at the same time points) ±SD. (**b**) Blue native PAGE analysis of mitochondria isolated from wild-type (**1,2**) and *aim23Δ* **(3,4)** strains. Approximately, 100 µg of mitochondria were digitonin-solubilized and applied on 4–10% Blue native PAGE. After electrophoresis, the gel was sliced for tracks that were additionally stained with Coomassie G250 (1, 3) or for CIV activity (2, 4). (*) and (**) signs for CIII(2)CIV(1) and CIII(2)CIV(2) positions, respectively; supercomplexes, CV, and CIV positions are indicated.

We previously characterized the growth of *S. cerevisiae aim23Δ* strain in liquid YPEG media and showed that pronounced lag-phase was not mediated by overgrowth of the inoculum fraction carrying secondary compensatory mutations [9]. To determine the mechanism underlying the adaptation of the strain with deleted *AIM23* gene, we decided to test whether mitochondrial dysfunction in this strain, caused by imbalanced mitochondrial translation at early stages of respiratory growth, is later compensated by restoration of OXPHOS (oxidative phosphorylation system) activity. As we demonstrated previously, translation of COX1, COX2, and COX3 mRNAs was most affected by the absence of Aim23p, while translation level of CytB was retained as in the wild type. We hypothesized that adaptation to respiratory growth described above was mediated by the restoration of cytochrome c oxidase activity. Therefore, we tested the cytochrome c oxidase activities in wild-type and *aim23Δ* strains’ mitochondria after 6, 48, and 72 h of cultivation on YPEG medium (before and after adaptation) (Figure 1a).

Thus, as it could be seen on Figure 1a, the activity of cytochrome oxidase in *aim23Δ* strain before adaptation to respiratory growth was lower than 10% of that in wild type. Nevertheless, after adaptation (72 h time point), when yeasts with deletion grew in the same manner as wild type cells, the activity of CIV did not completely restore and comprised nearly 40% from that in wild type.

### 3.2. Deletion of AIM23 Completely Prevents Supercomplexes Formation, Which Does Not Restore after Adaptation to Respiratory Growth

As it was shown previously, complexes III and IV (further referred to as CIII and CIV, respectively) of *S. cerevisiae* inner mitochondrial membrane are associated in supercomplexes with major stoichiometry CIII(2)CIV(1) and CIII(2)CIV(2) [19]. To test whether deletion of *AIM23* affects these supercomplexes formation before and after adaptation to respiratory growth, we performed the Blue native electrophoresis of digitonin-solubilized mitochondria purified from wild-type and *aim23Δ* strains after 4 h and 72 h of growth on YPEG media (Figure 1b).

In our experimental conditions, we didn’t see any supercomplexes with CIII or CIV either before or after adaptation of *aim23Δ* strain to respiratory growth compared to wild-type strain.

### 3.3. Tma19p is Overproduced During Adaptation of Aim23p-Deficient Yeasts and is Indispensable for Its Respiratory Growth

**Figure 2:**
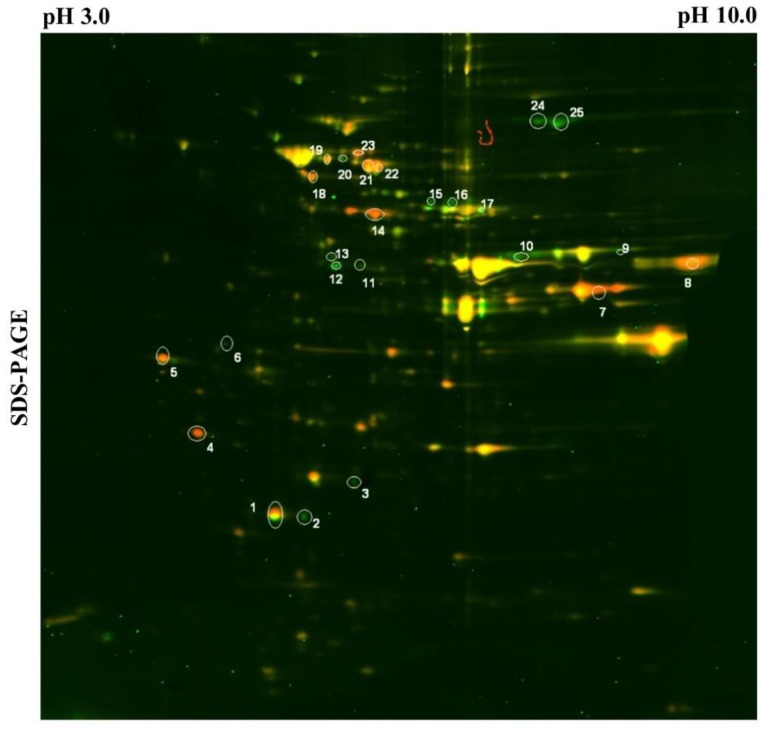
2D-DIGE analysis of wild-type (green) and *aim23Δ* (red) strains’ proteins after 24 h of growth on YPEG. Spots sliced for identification are marked.

As we showed previously, *S. cerevisiae aim23Δ* strain demonstrated retarded growth on media with glycerol as non-fermentable carbon source. The prolonged lag-phase followed by wild type–like exponential phase allows supposing some adaptation mechanism, which could include overproduction of one or more unidentified proteins that help mitochondria to restore its function. Trying to identify these hypothetical proteins, we performed differential proteomic 2D analysis (2D-DIGE) of whole cell lysates prepared from wild-type and *aim23Δ* strains, the latter being in course of adaptation (24 h), followed by mass-spectrometry identification of differentially expressed proteins (Figure 2, Table 1). One of the highest differences in expression level was identified for cytosolic Tma19p protein, which was overproduced in *aim23Δ* strain by more than 11 times compared to wild-type strain.

**Figure 3:**
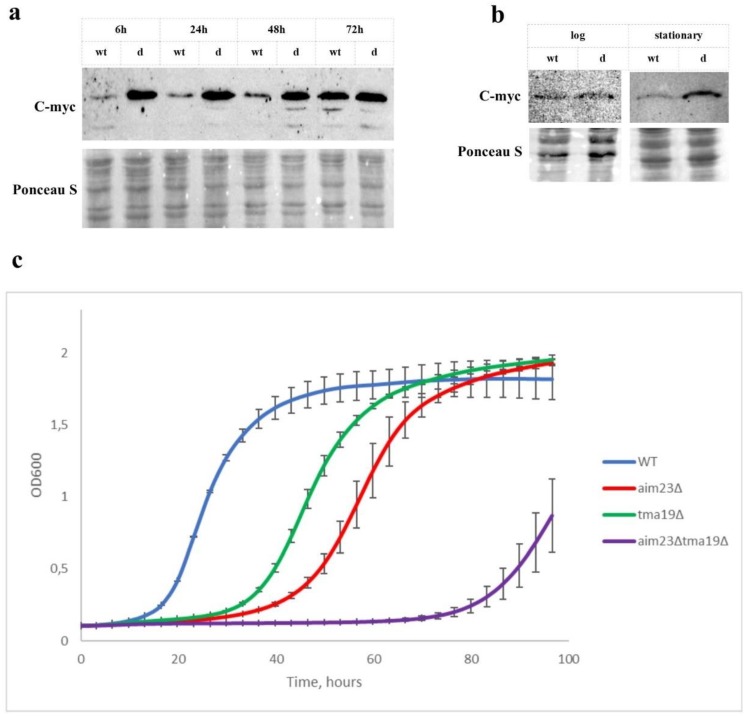
Tma19p is overproduced in *aim23Δ* strain during adaptation to respiratory growth. (**a**) Western-blot analysis of Tma19p steady-state levels at different time points of wild-type and *aim23Δ* strains grown on YPEG. In both strains, Tma19p was C-myc-tagged. Yeasts were grown overnight at YPD, washed twice with sterile water; for 6 h time point, amounts of cells equivalent to 1 OD were resuspended in 5 mL of YPEG and incubated at 30 °C with vigorous shaking; for other time points yeasts were inoculated into 10 mL of YPEG up to OD_600_ = 0.05 and incubated at the same conditions for indicated time points. **(b)** Steady-state levels of Tma19p at log-phase (OD_600_ ~1) and late stationary phase (OD_600_ ~5) of wild-type and *aim23Δ* strains’ growth on YPD. In both strains, Tma19p was C-myc-tagged. **(c)** Growth curves of wild-type, *aim23Δ*, *tma19Δ*, and *aim23Δtma19Δ* on YPEG medium. Yeasts were grown overnight at YPD, washed twice with sterile water, and inoculated into 1 mL of YPEG in 24-well plates at OD_600_ = 0.01; OD_600_ measurements were performed for 96 h every 15 min; experiments were repeated three times, mean values +/− SD are presented.

To confirm this observation, we created wild-type and *aim23Δ* yeast strains with C-Myc-tagged Tma19p and analyzed the level of this protein at different adaptation stages by western blot (Figure 3a). Using this approach, we showed that Tma19p amount was indeed significantly increased in *aim23Δ* strain at early stages of respiratory growth compared to wild type. This difference in Tma19p synthesis was pronounced at 6, 24, and 48 h of cultivation in YPEG medium. At 72 h time point, Tma19p levels in wild-type and *aim23Δ* strains were comparable to each other. Additionally, we analyzed the steady-state levels of Tma19p in these strains grown on fermentable carbon source (Figure 3b). At early log-phase time point (after 6–8 h of cultivation, OD_600_ ~1) the amount of Tma19p were low and similar in both strains. At the late stationary phase (after 18 h of cultivation, OD_600_ ~5), the level of Tma19p in *aim23Δ* strain was significantly higher than in wild-type. Thus, Tma19p overproduction at early growth phases of *aim23∆* correlates with mitochondrial function.

To check the significance of the Tma19p for *aim23Δ* strain adaptation to respiratory growth, we created two strains, *tma19Δ* and *aim23Δ/tma19Δ*, and tested their abilities to growth on non-fermentable carbon source (Figure 3c).

Deletion of *TMA19* gene led to some lag-phase prolongation which was, however, less pronounced than in the case of *AIM23* gene deletion. At the same time, double-deletion resulted in the dramatical increase of lag-phase up to 68–70 h. Moreover, *aim23Δtma19Δ* strain did not reach the OD600 values comparable to wild type, *tma19Δ*, and *aim23Δ* strains (Figure 3c) on the observed time scale.

### 3.4. Deletion of TMA19 Gene is Suppressed by Addition of Antioxidants in Both tma19Δ and aim23Δtma19Δ Strains

**Figure 4:**
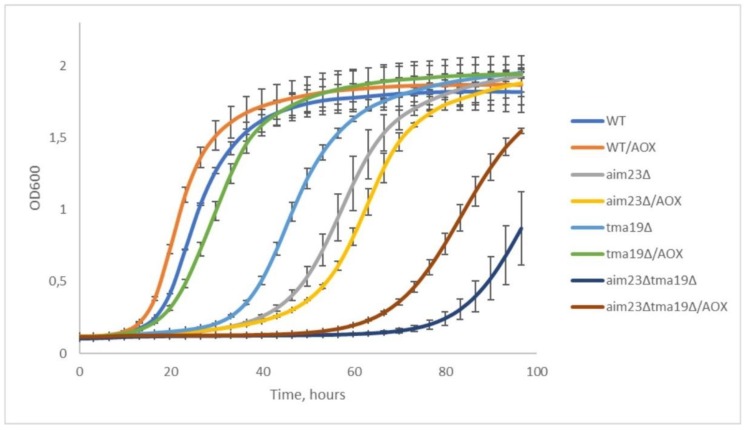
Addition of antioxidants partially rescues the prolongation of lag-phase caused by *TMA19* gene deletion. Growth curves of wild-type, *aim23Δ*, *tma19Δ*, and *aim23Δtma19Δ* on YPEG medium with and without antioxidants (AOX) (5 mM N-acetylcysteine and 1 mM α-tocopherol). Yeasts were grown overnight at YPD, washed twice with sterile water, and inoculated into 1 mL of YPEG with or without antioxidants in 24-well plates at OD_600_ = 0.01; OD_600_ measurements were performed for 96 h every 15 min; experiments were repeated three times, mean values ± SD are presented.

Tma19p is known to associate with mitochondria surface at some stress conditions [20]. We hypothesized that disturbance in mitochondrial-encoded cytochrome c oxidase subunits’ translation led to some functional disbalance of OXPHOS, which consequently resulted in increased ROS (reactive oxygen species) production. In this case, hyperproduction of Tma19p could prevent mitochondria from disastrous action of ROS and give mitochondria some time, which is vitally necessary for synthesis and assembly of required quantities of cytochrome c oxidase. If so, presence of antioxidants should shorten the lag-phase of *aim23Δtma19Δ* strain on respiratory media. To verify this assumption, we measured growth curves for wild type, *aim23Δ*, *tma19Δ*, and *aim23Δtma19Δ* in the YPEG medium in comparison with the same medium supplemented with well-known antioxidants—α-tocopherol and N-acetylcysteine (Figure 4).

Our data clearly showed that addition of antioxidants significantly shortened the lag-phase of strain with double-deletion, while low or no effect was detected in case of wild-type and *aim23Δ* strains. It should be noted that addition of antioxidants restored the respiratory growth of *tma19Δ* strain almost to the wild-type levels, which additionally indicated the role of Tma19p in oxidative stress response.

## 4. Discussion

Mitochondrial translation is generally organized in prokaryotic manner but has some unique features, especially in ribosomes’ structure and translation control. Most of the known mechanistic differences in translation regulation are related to translation initiation steps. Thus, initiation factor 3 in mitochondria shares a little homology to bacterial IF3. Probably due to this, it has not been identified in well-studied organisms such as *C. elegans* and *S. cerevisiae* for many years. Recently Aim23p was identified as mtIF3 in budding yeasts, and it was characterized biochemically and physiologically [8]. Interestingly, Aim23p acts not as bacterial or eukaryotic IF3 as it is not dispensable for mitochondrial translation. Moreover, its absence impacts differentially on the translation of different mitochondrial mRNAs. The absence of Aim23p reduces translation of COX1 and COX2 mRNAs, but increases synthesis of Atp6p and Atp8p proteins. Phenotypically, deletion of *AIM23* gene does not lead to inability of yeasts to grow on non-fermentable carbon sources, but drastically increases the lag-phase of culture transferred to respiratory medium [9]. In this work, we studied molecular mechanisms underlying such adaptation of Aim23p-less yeasts to respiratory growth.

At first, we hypothesized that increased lag-phase of *aim23Δ* strain’s respiratory growth is caused by prolonged time required for the synthesis of mitochondrially-encoded complex IV components amounts, sufficient for complete restoration of cytochrome c oxidase activity. To test this assumption, we measured the activities of CIV before and after adaptation. At the early stage of adaptation, CIV activity comprised only 10% of wild-type, while after adaptation it consisted just near 40% of wild-type. These data are in good agreement with earlier works where it has been demonstrated that there is CIV threshold activity level enough for normal OXPHOS function [21,22]. We supposed that relatively low CIV activity could be compensated by more effective supercomplex formation and consequent improvement of OXPHOS efficacy due to substrate channeling effect. Surprisingly, in our experimental conditions we could not identify any supercomplexes either before or after adaptation in *AIM23*-deficient strain’s mitochondria. To our opinion, this could be the consequence of small amount of assembled CIV due to poor biosynthesis of COX subunits encoded in mitochondrial genome. This, however, has to be experimentally verified in future.

Next, we supposed that mechanism allowing cells to survive until CIV activity reaches values enough for respiratory growth might include some changes in yeast gene expression profile. 2D-DIGE analysis revealed two proteins that were highly overproduced in *aim23Δ* strain—Tma19p (11.06 times more than in wild type) and Ef1bp (28.92 times more). Tma19p, also named Mmi1p, is an ortholog of the mammalian translationally-controlled tumor protein (TCTP) and is known to associate with mitochondrial outer membrane under stress conditions [20]. Remarkably, mammalian TCTP binds to eEF1B and specifically antagonizes the eEF1B-mediated GDP/GTP exchange reaction in the elongation reaction of protein synthesis in higher cells [23]. Based on these facts, we assume that hyperproduction of Tma19p helps cells to survive until OXPHOS becomes ready for efficient work, while Efb1p overproduction is a consequence of increased synthesis of Tma19p and is intended to compensate the impact of high Tma19p concentration on cytosolic translation. We additionally checked the level of Tma19p by constructing wild-type and *aim23Δ* strains with C-myc tagged Tma19p and western-blot analysis at different time points of respiratory growth. Indeed, Tma19p was overproduced in *aim23Δ* strain compared to wild type, especially at early stages of adaptation. At stationary phase, levels of Tma19p were equal in wild-type and deletion strains, probably due to well-known accumulation of ROS in aged cells [24]. The difference in Tma19p levels between wild-type and *aim23Δ* strains could also be explained by its production in varying quantities at different stages of culture growth. By the fact, this assumption could not be applied to our data because comparison of Tma19p levels in wild-type and *aim23Δ* at the same phases of respiratory growth (e.g., 6 h wild-type versus 24 h *aim23Δ* and 24 h wild-type versus 48 h *aim23Δ* etc.) revealed the increase in Tma19p production in the strain with deletion. This supports the assumption that Tma19p levels likely did not correlate with the growth phase, and is in agreement with our hypothesis that the *AIM23* deletion is the cause of increased Tma19p levels. Additionally, we compared the levels of Tma19p in wild-type and *aim23Δ* strains grown on YPD medium at early exponentional phase and stationary phase. In the the conditions of exponentional growth and glucose repression (after 6–8 h of cultivation, OD_600_ ~1), the levels of Tma19p were low and comparable in both strains. In contrast, at late stationary phase (after 18 h of cultivation, OD_600_ ~5), when glucose repression turned off and cells entered the diauxic shift, the amount of Tma19p in *aim23Δ* strain was significantly increased in comparison to wild-type strain. This observation supports our assumption that Tma19p is hyperproduced in *aim23Δ* strain during adaptation to respiratory growth. The marked difference in Tma19p production in wild-type strain between late stationary phase of glycolitic growth and stationary phase of respiratory growth could be explained by the following assumption. The late stationary phase of glycolitic growth in our experimental conditions actually represents the diauxic transition in yeasts’ growth when mitochondria are still inactive and ROS production is relatively low [25], while the 72 h time point of respiratory growth corresponds to the late phase of respiratory growth when the carbon source is almost exhausted and the majority of cells are aged and produce a lot of ROS. Moreover, our results are in line with data from [20], where the role of Tma19p in cell aging was shown. Nevertheless, it should be mentioned that our data do not deny the possibility of prolonged life-time of Tma19p in stress conditions instead of its hyperproduction. This should be experimentally verified in the future.

The importance of Tma19p hyperproduction for adaptation was shown by construction of strain with double-deletion (*AIM23* and *TMA19*). This strain had significantly longer lag-phase in comparison to *aim23Δ*, and did not reach stationary phase OD_600_ values typical to wild type strain at the observed time scale. Addition of commonly used antioxidants N-acetylcysteine and α-tocopherol to the medium partially decreased the duration of double-deletion strain’s lag-phase. This latter observation could be interpreted as an indication of Tma19p role in yeast adaptation to imbalanced mitochondrial translation, i.e., prevention of harmful action of high ROS concentrations on cells.

Taken together, our results allow supposing the following model of *aim23Δ* strain adaptation. In the absence of Aim23p, after switching cells to non-fermentative carbon source medium and consequent cessation of glucose repression, mitochondrial translation is imbalanced. Due to this, correct assembly and functioning of CIV is impossible, which leads to the incorrect work of OXPHOS, possibly accompanied with an increased ROS production. The latter could be a danger signal for cell; thus, an emergency scenario is run that implies overproduction of mitochondria-protective protein Tma19p. High concentration of Tma19p gives cells a chance to survive until mitochondria synthesize and accumulate enough quantities of COX subunits for sufficient OXPHOS function. We assume that supposed mechanism could be not unique for “Aim-less” mitochondrial translation, but may be also realized in other cases of mitochondrial translation disturbance.

## Figures and Tables

**Table 1:** Identified proteins differentially expressed in wild-type and *aim23Δ* strains during adaptation to respiratory growth.

Spot Number	*aim23Δ* Versus WT Ratio	Protein Name	Spot Number	*aim23Δ* versus WT Ratio	Protein Name
1	2.06	Ahp1p	14	4.65	Hxk1p
2	0.39	NI *	15	0.47	Ino1p
3	0.27	Pst2p	16	0.87	Ino1p
4	11.06	Tma19p	17	0.78	Ino1p
5	28.92	Efb1p	18	2.47	Hsp60p
6	0.38	Tpm1p	19	3.35	Vma1p
7	3.58	Adh1p	20	0.65	Vma1p
8	1.44	Pgk1p	21	2.32	Ssb1p
9	0.38	Erg13p	22	3.1	Ssb1p
10	0.56	Eno1p	23	2.78	NI *
11	0.55	Gpd1p	24	0.39	Met6p
12	0.22	Sam2p	25	0.35	Met6p
13	0.26	Lys9p			

* NI, not identified.
